# Context and Cardiovascular Risk Modification in Two Regions of Ontario, Canada: A Photo Elicitation Study

**DOI:** 10.3390/ijerph6092481

**Published:** 2009-09-17

**Authors:** Jan E. Angus, Ellen Rukholm, Isabelle Michel, Sylvie Larocque, Lisa Seto, Jennifer Lapum, Katherine Timmermans, Renée Chevrier-Lamoureux, Robert P. Nolan

**Affiliations:** 1 Lawrence S. Bloomberg Faculty of Nursing, University of Toronto, 155 College Street, Rm 130, Toronto, ON M5T 1P8, Canada; E-Mail:lisa.seto@utoronto.ca; 2 School of Nursing, Laurentian University, 935 Ramsey Lake Road, Sudbury, ON P3E 2C6, Canada; E-Mails:erukholm@laurentian.ca (E.R.);slarocque@laurentian.ca (S.L.);ke_timmermans@laurentian.ca (K.T.); 3 Resources, Research, Evaluation and Development Division, Sudbury & District Health Unit, 1300 Paris Street, Sudbury, ON P3E 3A3, Canada; E-Mails:micheli@sdhu.com (I.M.);chevrierr@sdhu.com (R.C.L.); 4 Daphne Cockwell School of Nursing, Ryerson University, 350 Victoria Street, Toronto, ON M5B 2K3, Canada; E-Mail:jlapum@ryerson.ca; 5 Behavioural Cardiology Research Unit, University Health Network, NU 6N-618, 585 University Avenue, Toronto, ON M5G 2N2, Canada; E-Mail:rnolan@uhnres.utoronto.ca

**Keywords:** cardiovascular disease, place, cardiovascular risk modification, qualitative methods, health behaviour

## Abstract

Cardiovascular diseases, which include coronary heart diseases (CHD), remain the leading cause of death in Canada and other industrialized countries. This qualitative study used photo-elicitation, focus groups and in-depth interviews to understand health behaviour change from the perspectives of 38 people who were aware of their high risk for CHD and had received information about cardiovascular risk modification while participating in a larger intervention study. Participants were drawn from two selected regions: Sudbury and District (northern Ontario) and the Greater Toronto Area (southern Ontario). Analysis drew on concepts of place and space to capture the complex interplay between geographic location, sociodemographic position, and people’s efforts to understand and modify their risk for CHD. Three major sites of difference and ambiguity emerged: 1) place and access to health resources; 2) time and food culture; and 3) itineraries or travels through multiple locations. All participants reported difficulties in learning and adhering to new lifestyle patterns, but access to supportive health resources was different in the two regions. Even within regions, subgroups experienced different patterns of constraint and advantage. In each region, “fast” food and traditional foods were entrenched within different temporal and social meanings. Finally, different and shifting strategies for risk modification were required at various points during daily and seasonal travels through neighbourhoods, to workplaces, or on vacation. Thus health education for CHD risk modification should be place-specific and tailored to the needs and resources of specific communities.

## 1. Introduction

Cardiovascular diseases, which include coronary heart diseases (CHD), remain the leading cause of death in Canada and other industrialized countries, although mortality rates are declining [1]. Earlier diagnosis and improved medical management have contributed to longevity of people with CHD, and a corresponding decrease in major risk factors such as smoking has served to reduce its incidence [1,2]. International guidelines are based on the assumption that people at high risk for CHD benefit from healthy modifications to diet, tobacco use, exercise, and management of diabetes and hypertension [3–5]. A recent meta-analytic review noted that individually focused multiple risk reduction interventions result in small changes in target behaviours and have a low impact on morbidity and mortality [6]; nevertheless, the impact of these interventions is significant and independent of available medical therapy. Hence, the clinical importance of risk modification should not be underplayed. Two branches of research have explored the difficulties people face in making sense of and acting on risk reduction advice.

One stream of inquiry is concerned with *how* people receive and act on risk reduction advice. This work indicates that understanding and personalizing the notion of cardiovascular risk involves “lay epidemiology”, which incorporates local meanings, familial experience, and known exceptions to the purported health effects of risk factors such as smoking [7–9]. The in-between, or liminal, state of being “at risk” is problematic for those who feel healthy but must interpret the meaning of diagnostic indicators such as elevated cholesterol levels [10]. Once identified as “at risk”, people may strive to minimize and normalize their risk status by contextualizing it as part of the life course [11]. Women and men may assign different meanings and attribute different causes to CHD—for example, women may perceive themselves as more susceptible to breast cancer and men as more prone to CHD [8,12,13]. Furthermore, it is difficult to change established routines and, over the longer term, many people struggle to adhere to healthy lifestyle recommendations [14–17].

Another research focus is on *where* people receive and act on risk reduction advice, because sociodemographic position and geographic location form circumstances that support or undermine health [18]. Health regions in Canada with higher levels of heart disease mortality also have greater prevalence of smoking and obesity than the national average [19]. Ontario is among the provinces with the lowest levels of smoking-attributable mortality and prevalence of CHD risk factors [2]. Previous research indicated the province has regional differences in the prevalence of modifiable risk factors, with higher risk, morbidity and mortality in some areas of northern Ontario [20]. Grace [21] found that, among a sample of Ontario residents with cardiovascular disease, diabetes, or two or more cardiovascular disease risk factors, and who subsequently received risk modification information through a telephone counseling intervention, the frequency of exercise was lower in residents of northern Ontario than in urban residents of southern Ontario. Furthermore, even within regions, risk factors are inequitably distributed. For example, francophone communities in northern Ontario have lower average incomes than anglophone Ontarians and higher prevalence of risk factors, especially cigarette smoking; on average this population is older and reports higher incidence of CHD [22,23]. CHD risk factors are also more prevalent in disadvantaged inner city residents of Toronto than in the general urban population, and inner city residents encounter greater barriers to risk modification [24]. Physical inactivity is more prevalent in the Toronto area than some northern areas of the province [2].

The importance of both approaches lies in their potential to lead us beyond individuated approaches to health behaviour change. For example, “lay epidemiology” draws attention to local and collectively constructed knowledge about heart disease and health behaviour [7–9]. These shared understandings are part of social connectivity and positioning, which in turn may be threatened by shifts of identity posed by health risk or health-related behaviour change. Attention to patterns in particular geographic regions highlights the role of local infrastructures, climate and economic conditions [18]. These contextual circumstances play out differently for sociodemographic groups within the same region because they are afforded different opportunities, barriers and resources for health. Thus, within both streams of research, there is indication that an ecological lens is necessary in understanding the fate of people’s risk reduction goals.

### 1.1. Purpose

There is a need to illuminate the complex interplay between geographic location (place), sociodemographic position, and people’s efforts to understand and modify their risk for CHD. We undertook a qualitative study to examine and compare experiences with cardiovascular risk modification in two selected regions: Sudbury and District (northern Ontario) and the Greater Toronto Area (southern Ontario). This was linked with a larger trial (led by RPN) that tested a risk reduction telephone counseling intervention for individuals at high risk for CHD in three regions of Ontario. Trial participants’ risk for cardiovascular disease was determined based on the Framingham algorithm, using a standardized telephone assessment interview and physician referral form [25,26]. Individuals at elevated risk for cardiovascular disease (i.e., two or more risk factors) were invited to participate in the trial, including those with an existing diagnosis of atherosclerotic heart disease. The purpose of our associated qualitative study was to understand the social and material conditions of health behaviour change from the perspectives of people who had been made aware of their high risk for CHD and had received information about cardiovascular risk modification.

## 2. Data Collection and Methods

The methodological foundations of critical ethnography were well suited to this inquiry, because they encourage attention to the social organization of everyday activity [27,28]. We augmented focus groups and in-depth interviews with photo-elicitation techniques [29–31]. First, focus group discussions encouraged 4–6 participants to share insights gained through learning about their risk for CHD, setting personal goals, and making lifestyle changes to modify their risk factors. Following focus groups, disposable cameras were distributed and participants were instructed to take pictures of everyday scenes or objects that influenced their health or represented their experiences with risk modification. This offered the opportunity to creatively and dramatically *show* us some of the geographic, social and practical influences on health behaviour change. After the photographs were developed, individual, hour-long, semi-structured interviews were focused on the images to elicit in-depth discussion of the issues they represented. That is, the photos themselves were not analyzed; rather, they were used as a focal point within the interviews. In many cases, participants were able to reflect on habitual and infrequently acknowledged dimensions of their everyday lives as they considered what to photograph, created photographic images, and reviewed images in conversation with an interested and active listener [29–31]. Photographs were digitally anonymized by blurring facial features. Signs identifying businesses or restaurants were also blurred and replaced with representative generic terms such as “Fast Food”.

### 2.1. Recruitment

Research Ethics Board approval was obtained from the University of Toronto, Laurentian University, Sudbury & District Health Unit, and all hospitals that assisted with recruitment for the intervention study. Participants were recruited from Toronto (a densely populated urban metropolis) and Sudbury (a mid-sized city surrounded by a sparsely populated rural area). We sent letters of invitation to those who were registered in the larger intervention study sample. Sudbury and the surrounding rural area are home to clusters of French-Canadians, so we sought to include members of these communities by sending a French letter of invitation to the few participants in the larger intervention study who identified French as their first language. We also recruited francophone participants from a local cardiac rehabilitation program, because its participants were similarly aware of their risk factors and had received information about risk modification. Interviews and data analysis for each subgroup were conducted separately by locally situated research sub-teams; francophone subgroup data collection and analysis were done in French. Analysis was concurrent with data collection, and sub-teams communicated throughout the study using email, teleconferences, and intensive, one or two-day site meetings.

### 2.2. Theoretical Foundations and Analysis

Focus group discussions and interviews were professionally transcribed. Reflexive discussions and observations about the data were shared within and between sub-teams. The photographs were scanned and catalogued so that they could be referred to during analysis, but they were not individually analyzed. After several meetings and readings of interview data, a coding scheme was developed by consensus for all groups to use when coding in QSR NVivo7. The codes were translated and some new codes added by the francophone sub-team to capture the experience of this subgroup.

Analysis was theoretically informed by social theory, which finds praxis in critical ethnography [27,28]. We drew on de Certeau’s [32,33] concepts of place and space to refine attention to the interplay between everyday contexts and health behaviours. *Place* denotes the locations, physical or social, that people occupy or pass through in their daily rounds of activity [32,33]. Activities or practices make places into meaningful *spaces*. Space is fluid and it constantly transforms with the changing context; spatial stories reflect this ambiguity [32,33]. In telling about their daily practices, people guide the listener through their *itineraries,* or travels through the practiced spaces of the everyday [32,33]. In their spatial stories, participants described their embodied movements through the microgeographies of their everyday worlds, claiming locations and situating themselves within them in meaningful ways. These habitual practices become embedded in place, so that context itself begins to call forth behaviours.

As our analysis deepened, we saw that the process of adding or changing activities for CHD risk reduction problematizes spatial practices. People must find or make new space for exercise and new dietary practices, and they must resist the call to act in habitual ways within existing spaces. In so doing, they change the character of their everyday worlds. Furthermore, in changing the spatial practices for themselves, individuals may change for others the meaning of communally occupied social spaces. We shared our findings in a plain language newsletter that was sent to the study participants and others; the newsletter was translated into French for the francophone community.

## 3. Results

### 3.1. Sample Characteristics

Of the 39 study participants, 21 were from Toronto and 18 were from Sudbury and Districts. Of the Sudbury subgroup, 12 were anglophone and six were francophone. There was equal distribution of men and women in each subgroup. Immigrants to Canada comprised 33% of Toronto participants and 17% of the Sudbury anglophones. Sudbury francophones were all born in Canada. Most participants were married or lived with someone, including 75% of the Sudbury anglophones and 100% of the Sudbury francophones, but only 57% of the Torontonians.

Focus groups, interviews, and over 340 photographs covered a wide range of self selected topics, including food; cigarettes; medication; locations for exercise; televisions and computers; nature, outdoors and weather; and family, friends and social occasions. All participants described efforts involved in initiating and maintaining recommended lifestyle changes, but different patterns of barriers and resources were seen between regions and subgroups.

### 3.2. Places, Spaces and Itineraries: Exemplars and Ambiguities

Analysis drew on de Certeau’s [32,33] concepts of place, space and itineraries as a lens to study the interplay between contexts and individual health activities. For example, this single woman described the physical layout of her open-concept kitchen and living room as a physical *place*. The meaning of this area of her home shifts ambiguously throughout her story about it. The open-concept arrangement was a “selling feature” that initially attracted her. The kitchen became a healthy or unhealthy *space* as its meaning was altered by the foods she consumed and her own “frame of mind”:

Participant: Okay, my kitchen. It can be both positive and negative. There are some days where I’m in there just looking for any kind of food. And it’s very negative because if the junk [snack food] is in there, I can stand at the counter, watch TV and eat…Interviewer: Okay. It’s an open-concept type area? Participant: Exactly, open-concept. When I bought my apartment that was the selling feature. It’s also good because that’s where I can make more healthy foods…I can have more vegetables and if I’m in the right frame of mind, it’s great to be in there. You chop the veggies and make something good.

**Figure 1.**
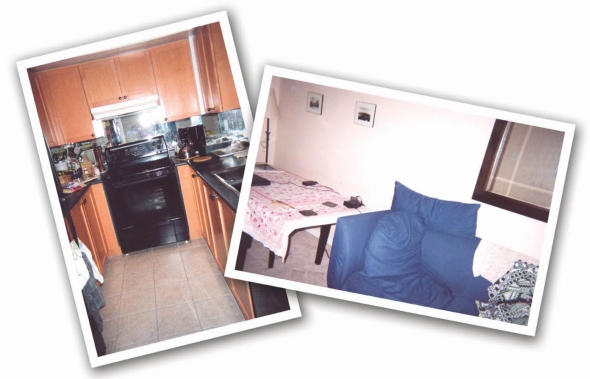
The ambiguous spaces of an open-concept kitchen and living room as the location for healthy or unhealthy food consumption and television viewing. Participants described habitual activities that created meaningful spaces within everyday locations.

Her account is a spatial story; it connects places in her home by telling the movements of her practicing body. The ambiguity of her kitchen as a space for practicing “positive or negative” eating habits is further seen in the open-concept connection to the living room. From her kitchen she can see the TV and be distracted from noticing her intake of “junk” or snacks. In the living room is her sofa, where she also spends time watching TV (see Figure 1).

Participant: But yeah, that’s where I sit and watch TV and I eat all kinds of nonsense. It’s really a bad place, um because I’m not out exercising; I am just watching TV… Interviewer: Okay. How do you feel sitting here, how do you feel in this area? Participant: Um, it’s funny because you would think it would be a negative feeling. But it’s such a relaxed feeling; it really is home in that way…It’s just so comfortable. I don’t worry about anything there; it’s my little corner of the world.

Within her spatial story, she describes her habitual *itineraries* through the microgeography of her home. Routines of comfort conflict with her consciousness of heart health in diet and exercise, problematizing long established spatial practices. The TV is a focal point for some of her itineraries; she can view it from her kitchen, as she collects snack food, or from her livingroom sofa. The everyday locations of home and neighborhood became spaces of ambiguity and struggle while participants sought to change their health behaviours.

### 3.3. Place and Access to Health Resources

Toronto participants described easy access to a wide variety of health resources such as gyms, family doctors, cardiac specialists, and health promotion programs. A higher concentration of health resources is typical of larger cities. Some participants spoke of being able to reach most destinations by walking or taking public transit. However, many also remarked on the lack of parks or green areas in the city. They traveled some distance to natural settings for exercise and for the stress release they associated with beautiful parks or out-of-town retreats. Those with higher incomes could afford to pay for exercise equipment or health club fees, but then had to make appropriate space for use of these resources in their homes or crowded schedules. One man showed us a photograph of his cluttered garage: “*It shows where I buried my treadmill…If you look closely, you can see the dust on it…”*

Torontonians with lower incomes could not afford such resources, however, and some participants found the city did not easily yield space for safe exercise. One man had lost weight by establishing a new habitual practice of jogging through his neighborhood. He explained the perils of running or biking in the city:

I find some times running—I don’t know what it is but… there is a definite percentage of population who, for some reason, just don’t like us. And if they’re in a car, and if they can somehow cut you off [when you are] either running or on a bike, they’ll do it.

An elderly man proudly showed us a photograph of himself working out at an upscale gym. He had immigrated to Canada in his later years and did not qualify for sufficient public pension funds to support him in retirement. He was employed fulltime at the gym, where he was allowed to make full use of the facility for his own fitness—after a day of cleaning floors, laundering towels, and tidying change rooms. This form of access was precariously based on his continued ability to do physical work, because he could not afford to pay membership fees.

**Figure 3.**
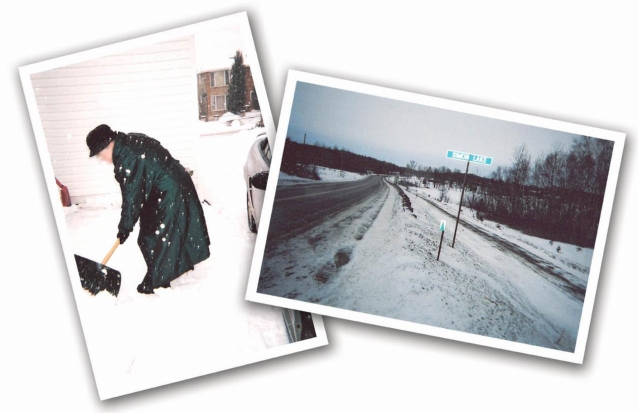
A Sudbury woman shovels snow to clear her driveway. A car is a necessity in less densely populated areas. Heavy snow and icy roads are unsafe and impede road travel.

Sudbury anglophone participants described harsh, long winters that limited outdoor exercise and travel to health-related appointments. Cars were a necessity for travel to more widely dispersed health resources. Those who lived on the outskirts of the city needed consistent access to a well maintained vehicle—something that many could not easily afford (see Figure 3). One man recounted his worries about car breakdowns during a period of financial hardship:

…since I was living 20 minutes from the nearest shop or repair shop or trucking centre or so—and my car was almost 16 years old…I never took it to the repair shop in 12 years. I did most of the work myself. What I couldn’t do, I hoped it wouldn’t break down (laughs)…

Those with additional chronic health problems such as arthritis found it difficult to carry out basic daily tasks under such harsh climatic conditions and also had to contend with clearing snow. This woman lived alone and described her struggles with the snow:

Well I can only do it for a very short time and then I am really exhausted and I know that if I kept on maybe I would take a heart attack.…And then the asthma is in there too, you know…

Many Sudbury anglophone participants also spoke of difficulties in finding health providers such as a family doctor or cardiac specialist. Those who had access to consistent health care providers felt “lucky” to have them. Gyms and indoor pools were not as numerous and were more dispersed than in Toronto. A few participants owned exercise equipment such as treadmills so they did not have to leave home to work out in winter. In the summer months, several anglophones took advantage of easy access to outdoor sports and nature-oriented activities.

Sudbury francophones described similar struggles with climate and access to health services. However, they had additional problems with finding French language services. This woman describes problems faced by residents of her small community some distance from Sudbury:

Oui, déjà il avait un docteur, le Dr. [X] était là. Aujourd’hui, son bureau au Dr. [X], est ouvert juste 2 jours par semaine. Ça fait longtemps qu’il essaye de prendre sa retraite puis on est pris pas de médecin. On n’est pas capable d’avoir de service, fait que, il demeure à Sudbury à présent puis il vient ici 2 journées par semaine pour voir nos gens. Parce que [X] c’est beaucoup des gens âgés. Ils sont accoutumés à lui et puis c’est pareil, ils n’ont pas les moyens, il y en a qui n’ont pas d’auto, ils ne conduisent pas.

[Translation: We had [Family Physician] he was a full-time doctor. . . . Today, the [Family Physician]’s office is open only two days a week. He’s been trying to retire for quite a while, and we’re stuck without a doctor. We can’t get treatment, because he lives in Sudbury now, and he comes here two days a week to see patients here. Because in [town] there are a lot of old people. They’re used to him, and then it’s the same thing, they haven’t got the money, some of them don’t have a car, they don’t drive.]

Thus, those who lived in close-knit francophone communities outside Sudbury had to circumvent the entanglement of multiple barriers, including severe weather, long-distance travel for primary or specialized health care, and limited availability of French-speaking Family Physicians, or *any* Family Physicians for that matter.

### 3.4. Time and Food Culture

Urban participants described exposure to a close concentration of advertising, “fast foods” and sedentary entertainments that created constant opportunities to indulge in risk behaviours. Those who were employed described the stress and time pressure of commuting as significant barriers to incorporating new health behaviours into everyday routines. Women, and some retired men, described additional time constraints posed by caregiving responsibilities for children, grandchildren or parents. Retirees explained that their new-found luxury of time supported their efforts to change; this man contrasted his life with that of someone in middle years:

…there are other priorities, they’ve got to make a living, and their boss says you stay till ten o’clock tonight, there’s this report, whatever. And, or you’ve got three children at home, and you have to, you know, you have to see to their needs.

Torontonians described lives that were temporally, spatially, socially and even commercially “crowded”. They turned to processed or “fast” restaurant-prepared food in efforts to reduce time spent in meal preparation, and to make space for pleasure and stress release.

In contrast, Sudbury participants found that loneliness and isolation were linked with time in the form of seasonal weather conditions. The winter months brought heavy snow, which even impeded travel for short distances, especially for those who lived outside the city itself. In isolated circumstances, indoor pastimes such as TV watching and computer games did not fully address the need for social companionship. Several Sudbury participants regularly met friends at “fast food” restaurants to socialize, yet the health benefits of social connection can be undermined by indulgence in inexpensive treats, as this man explains:

We have friends that go to [coffee and donut chain], practically every night, so once in a while we’ll go there as a group. And that’s a good experience, because we sit and chat. But if you overdo any of this stuff, that’s not good.

Fast food restaurants became spaces of tension, where the wellbeing associated with friendship and conversation was concurrent with worry about consumption of processed foods.

Similar issues were extensively discussed by francophones. Most regarded their francophone identity and group cohesion as unique and important, particularly given their history as a marginalized group in northern Ontario. Socializing with other francophones was closely associated with feasting on good-tasting foods that were not always heart healthy, as this participant explained:

On est en général, du monde qui a bien de la misère à rester enfermé, surtout l’hiver. Puis on aime ça sortir puis on va, comme on a mentionné a des restaurants ou des places que il y a beaucoup de chance a avoir du cholestérol ou trop de sel ou trop de sucre ou quelque chose d’autre, ça fait qu’on a une bonne chance d’avoir du trouble avec notre santé parce que nos habitudes de vies sont plus en général plus agressives que d’autre monde.

[In general, we’re people who have a lot of trouble staying cooped up, especially in winter. We like to go out, so we go places, like, we mentioned restaurants or places where there’s a good chance we’ll have cholesterol or too much salt or too much sugar or something else…Our lifestyle habits are more aggressive than other people’s]

Giving these foods up was hard because it decreased the pleasure of social events and changed a valued part of traditional practices that some regarded as central to their culture. Furthermore, four of the Sudbury francophone participants smoked and found it difficult to quit, especially when close friends or family members still smoked. The emphasis on family cohesion and support made it difficult to change shared activities. Intimate, private spaces were simultaneously a refuge and barrier for this subgroup.

### 3.5. Itineraries

Participants moved through several places in the course of daily activities, creating chains of spatial activity, or *itineraries* [32], that connected several locations over brief periods of time. These daily rounds offered a shifting array of challenges to and opportunities for health. Contradictory calls and practices were encountered throughout these travels. This Torontonian exercised regularly at a nearby running track. However, in returning to his home, he always passed a local restaurant that served his favourite breakfast. Sometimes he gave in to temptation, but reminded himself that these foods might not be best for his health:

They serve more bacon and eggs than any place in Metropolitan Toronto and it’s a fabulous greasy spoon restaurant. It is a detriment to me because sometimes when I’m coming back from the high school track, I have to go by this plaza on Saturdays and pick up some (whispering) big links of sausage and scrambled eggs which really is not good for me, and yet, maybe once a week or once every two weeks.

His anecdote indicates that people travel through multiple places on an everyday basis. Many of these locations are well-practiced spaces where habits are called forth as temptations, even as good intentions are formed and practiced at other sites. He worked to alter the frequency of stops at the restaurant, reducing his intake of high fat breakfasts, while occasionally giving in to nostalgia.

Similarly, participants from all sub groups spoke of challenges encountered when they were *dis-*placed from their new health behaviours while on vacation or travelling on business. This Sudbury francophone man described a complex interplay of meanings connected with culture, vacationing, and eating:

Bien, les français aiment manger. Les français aiment sortir et voyager qui est une peut-être une mauvaise combinaison parce que quand on sort et on voyage on sait pas tout le temps que c’est qui s’en vient sur notre assiette...et j’ai eu des expériences que, ou le mangé n’était pas tout à fait maigre, aussi bon pour moi et je pensais, donc uh, vu que les canadiens français aiment beaucoup voyager et manger, c’est une combinaison qui devrait être, qui devrait nous mettre sur nos gardes.

[Translation: Well, the French like to eat, the French like to go out and travel, which is maybe a bad combination, because when you go out and you travel, you don’t always know what’s going to be on your plate… and I’ve had some experiences where the food wasn’t quite as lean, quite as good for me as I thought, so since French-Canadians love to travel and eat, it’s a combination that should put us on our guard.]

While on holiday, many participants also permitted themselves a full break from dietary restrictions and risk modification. Thus, the interplay between place and individual health behaviour may constitute opportunities or challenges to creative change. Although meaningful places call people to resume or continue habitual activities, both of the above accounts suggest a new awareness of spatial practices, and ongoing efforts to incorporate new health-related meanings.

## 4. Discussion

### 4.1. Place and Access to Health Resources

Participants in two geographic regions of Ontario reflected on the challenges to CHD risk modification encountered in the places and activities of daily life. Patterns that constrain and support CHD risk modification manifested differently between geographic regions; they also differed between social groups within the same region. Our work may explain why Grace and colleagues [21] found that among Ontario residents with high risk for CHD, those from northern Ontario exercised with less frequency than urban or rural residents. Reliance on personal transportation, difficult access to gyms or outdoor exercise sites, low income, and harsh winter conditions all combined to limit opportunities for healthy activity levels among the Sudbury and Districts participants. In contrast to King and her colleagues [13], who found that rural Alberta residents believed their closeness to nature and their physically demanding work protected them from heart disease, our northern Ontario participants seemed more ambivalent about their natural surroundings. Although prevalence of physical inactivity is increasingly lower in the Sudbury & Districts region, isolation, severe weather, and stress associated with dangerous road conditions were believed to be detrimental to heart health. Limited public transportation further constrained access to health resources in Sudbury and Districts.

Effective health education for CHD risk modification should be place-specific, recognizing the limitations imposed by climate, low population density, language, and inadequate access to health resources, including shortage of primary care providers. Although summer activities such as hiking or trail biking were enjoyed by some Sudbury participants, outdoor winter sports were less frequently mentioned. The isolation or monotony of winter may attract community members to indoor activities and programs, although the cost of gyms or fitness classes was a concern to many participants in this study. Improved infrastructures for the region, including public transportation and low-cost community recreation programs, would increase options for risk modification.

### 4.2. Time and Food Culture

In both regions, participants struggled to avoid processed foods or unhealthy food items; however, many encountered social barriers that speak to the centrality of food in creating congenial space and group cohesion. This is consistent with findings of other qualitative studies [34,35], but there has been little research to date that illuminates the importance of traditional foods to cultural identity in francophone Ontarians. Immigrant Torontonians also remarked on their difficulties in eliminating high-fat or starchy traditional food items. As a team, we noticed how this phenomenon tended to problematize cultural identity for participants once they had been sensitized to the role of diet in cardiovascular disease. The complexities of dietary change extend into personal identity and social positionality. In food preparation, the “at risk” person may feel compelled to abandon the habitual “gestures of tradition” [36] (p. 210) and customary ingredients, and instead employ unfamiliar culinary approaches and food items. While this may inspire creation of hybrid diets with transnational exposure to new cuisines or information, it also may serve to subjugate valid traditional knowledge of food and nutrition [37]. Supportive social ties may erode when individual risk modification efforts create tensions around dietary preferences of close family or other cultural members.

Almost all participants described efforts to modify choices at fast food restaurants. Alter [38] recently determined that mortality and admission for acute coronary syndromes was higher in regions of Ontario with greater concentrations of fast food outlets; furthermore, this relationship was similar in high- and low-income communities. Our study indicates that there may be regional differences in the reasons why people gravitate to these outlets. Northern participants used these inexpensive restaurants as social space to connect with friends and ease the loneliness of isolation in wintertime. Urban participants often cited the lack of time to prepare meals, as well as the close concentration of multiple outlets. Although many such restaurants have added healthier menu items in response to advocacy from interest groups such as the Canadian Heart & Stroke Foundation, the majority of selections have poor nutritional value and contribute to risk for CHD [38]. Healthy public policy around food labeling and continued advocacy for improved fast food quality would further extend success in this domain. Community assessment and targeted health promotion can address unique regional needs. For example, more healthful meeting places and social activities could be identified or created within northern communities, and made available during evening hours.

### 4.3. Itineraries

Participants’ anecdotes highlighted their itineraries: short tours through the microgeographies of home and neighbourhood; longer, routine commutes to work or health services; or less frequent expeditions to vacation locales. These “travel stories”, as de Certeau [32,33] (p. 120) would call them, were replete with the minutiae of spatial practices and laden with references to the places that supported or impeded development of new health behaviours. New knowledge, distilled from the health sciences, became intertwined with and problematized habitual, practiced understandings of the everyday world.

The shared and social character of spatial practices further challenged many participants. However, these practices are not necessarily hardened into place; rather, there is constant exposure to newness, addition, and expansion. In achieving even the most subtle change, many participants navigated through a complex maze of the long-practiced habits of the everyday, the limits imposed by material circumstance, and the possibilities opened by new information. For participants, the major achievement of creating or altering an itinerary was in the transformation of place into new, personally meaningful spaces [36] that were perceived to contribute to risk modification. Initial health education for risk modification usually covers content such as nutrition, exercise, smoking cessation, and stress management. Follow-up support might include mapping itineraries with program participants to acknowledge the complexity of change and consider options for further creativity.

### 4.4. Strengths and Limitations of the Study

We used purposive sampling to recruit people from two regions of Ontario who were aware of their high risk for CHD, had been given information about risk modification, and had acted on this information. These participants could provide rich and detailed descriptions of contextual constraints and supports in the maintenance of health related behaviour change. Hence, their expertise strengthened the credibility of the study. However, this also resulted in recruitment of a sample that was deeply sensitized to the discourses of heart health. While this sensitization may have resulted in guarding of details around what some participants referred to as “cheating”, “laziness”, or “escapades”, we approached all interactions with participants in the understanding that change is inherently difficult, but barriers are not insurmountable. Most were open about such issues and supplied considerable detail.

The research was conducted by three sub-teams, one in Toronto, and two in Sudbury. The communication constraints created by distance should be weighed against the benefits of each sub-team’s familiarity with the local context during fieldwork and analysis. This was particularly important in working with the francophone subgroup in Sudbury. Intensive site meetings and frequent teleconferences facilitated reflexive analytic discussion, which was concurrent with data collection.

The use of photo-elicitation enabled most participants to tap into deeper insights and descriptions than might have been possible without the use of participant-generated images. However, photography may hold different meanings and challenges for people depending on social class, life experience and the subject matter [39]. Four participants declined to take pictures but remained willing to engage in rich discussion of their insights about everyday places and situations. One woman explained that she simply could not portray in a photograph the stressful situations she encountered due to illness of a family member. This suggests that use of photo-elicitation may not be appropriate for some topics and that some people may not be comfortable with this technique. We recommend that photo-elicitation interviews include additional questions such as: “Are there other places, things, circumstances you would like to discuss that could not be captured in a photograph?”

## 5. Conclusions

We sought to illuminate the complex interplay between geographic location (place), sociodemographic position, and people’s efforts to understand and modify their risk for CHD. This qualitative study drew on critical ethnography and concepts of place and space [32] to examine and compare CHD risk modification experiences in two selected regions: Sudbury and Districts (northern Ontario) and the Greater Toronto Area (southern Ontario). We also sought to compare the experiences of anglophones and francophones in Sudbury. The analysis was attentive to the places or physical locations of everyday life, as well as participants’ spatial practices or activities within these places. Participants’ photographs and interviews demonstrated how places are implicated within cardiovascular risk modification. All participants described the effort involved in initiating and maintaining recommended lifestyle changes, but different patterns of constraint and resources were seen between regions and subgroups. Access to supportive health resources was different in the two regions. A fast food culture was deeply entrenched in both regions, but it was anchored by different pressures. Even within regions, individual risk modification efforts were not fixed in place and time. Participants moved through several places in the course of daily activity, creating ‘itineraries’ or chains of spatial practice where constraints and supports to healthy behaviour shifted continually. Health behaviours were integral within these itineraries and were continuously challenged and reconstituted.

## Figures and Tables

**Figure 2.**
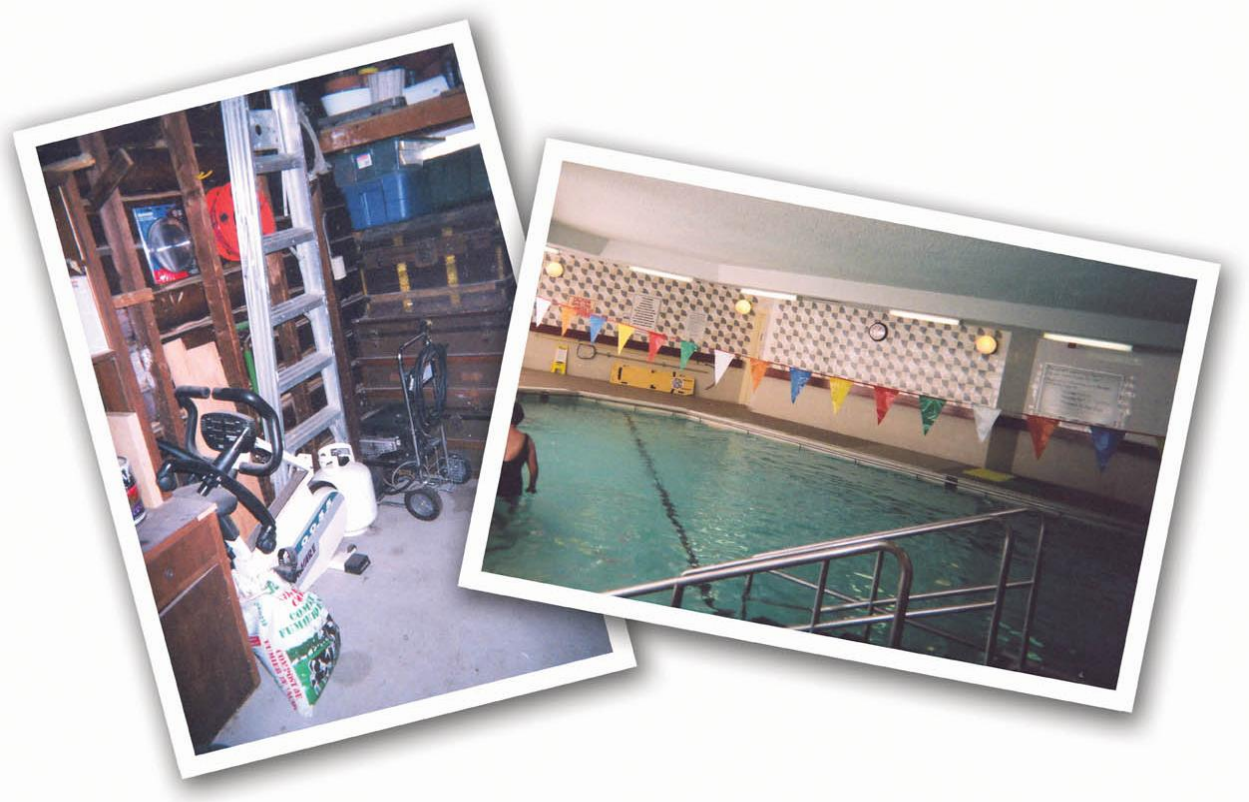
Finding places and making spaces for activity in the Toronto subgroup. One man could not find a space for his exercise equipment in his home, and finally stored it in his garage where it sat unused. A woman had arthritis, but exercised in a special heated pool at a rehabilitation facility that was close to her home.

**Figure 4.**
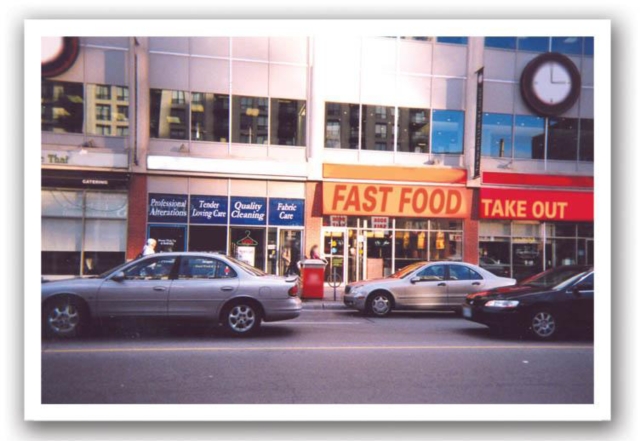
Rush hour traffic begins early in Toronto. These drivers pass fast food restaurants on their way home.

**Figure 5.**
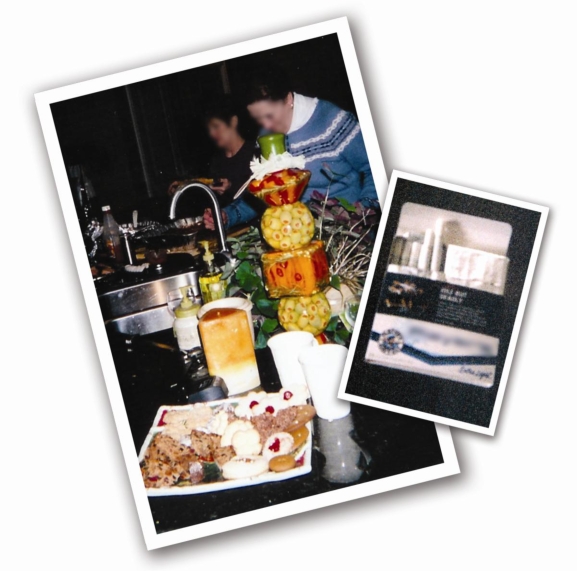
Nourriture et famille—A francophone family gathers for a special dinner with high fat and carbohydrate treats. Smoking is also a health issue among participants. Emphasis on group cohesion and supportive ties made changes in diet and tobacco use difficult for francophone participants.
